# Phenology largely explains taller grass at successful nests in greater sage‐grouse

**DOI:** 10.1002/ece3.3679

**Published:** 2017-11-28

**Authors:** Joseph T. Smith, Jason D. Tack, Kevin E. Doherty, Brady W. Allred, Jeremy D. Maestas, Lorelle I. Berkeley, Seth J. Dettenmaier, Terry A. Messmer, David E. Naugle

**Affiliations:** ^1^ Wildlife Biology Program University of Montana Missoula MT USA; ^2^ US Fish & Wildlife Service, Habitat and Population Evaluation Team Missoula MT USA; ^3^ US Fish & Wildlife Service Lakewood CO USA; ^4^ W.A. Franke College of Forestry and Conservation University of Montana Missoula MT USA; ^5^ USDA Natural Resources Conservation Service West National Technology Support Center Portland OR USA; ^6^ Montana Department of Fish, Wildlife & Parks Helena MT USA; ^7^ Department of Wildland Resources Jack H. Berryman Institute Utah State University Logan UT USA

**Keywords:** *Centrocercus urophasianus*, concealment, greater sage‐grouse, nest survival, phenology

## Abstract

Much interest lies in the identification of manageable habitat variables that affect key vital rates for species of concern. For ground‐nesting birds, vegetation surrounding the nest may play an important role in mediating nest success by providing concealment from predators. Height of grasses surrounding the nest is thought to be a driver of nest survival in greater sage‐grouse (*Centrocercus urophasianus*; sage‐grouse), a species that has experienced widespread population declines throughout their range. However, a growing body of the literature has found that widely used field methods can produce misleading inference on the relationship between grass height and nest success. Specifically, it has been demonstrated that measuring concealment following nest fate (failure or hatch) introduces a temporal bias whereby successful nests are measured later in the season, on average, than failed nests. This sampling bias can produce inference suggesting a positive effect of grass height on nest survival, though the relationship arises due to the confounding effect of plant phenology, not an effect on predation risk. To test the generality of this finding for sage‐grouse, we reanalyzed existing datasets comprising >800 sage‐grouse nests from three independent studies across the range where there was a positive relationship found between grass height and nest survival, including two using methods now known to be biased. Correcting for phenology produced equivocal relationships between grass height and sage‐grouse nest survival. Viewed in total, evidence for a ubiquitous biological effect of grass height on sage‐grouse nest success across time and space is lacking. In light of these findings, a reevaluation of land management guidelines emphasizing specific grass height targets to promote nest success may be merited.

## 1 INTRODUCTION

Environmental factors affecting influential demographic parameters are appropriate targets of management to promote habitat quality for species of conservation concern (Mills, 2007). For many birds, characteristics of nest sites that influence nest predation are of interest, as nest success is a key driver of population growth and predation is the primary cause of nest failure (Martin, 1993; Ricklefs, 1969). According to the nest concealment hypothesis, nests surrounded by dense vegetation should be more successful because they are more difficult for predators to detect or access (Martin, 1992; Martin & Roper, 1988). Furthermore, vegetative concealment may represent an attractive target for conservation action because it can often be managed, for example, through manipulation of herbivory by livestock.

Support for the nest concealment hypothesis is mixed. In a recent review and comparative analysis, 26% of 114 reviewed studies in open‐cup‐nesting songbirds supported an effect (Borgmann & Conway, 2015). Effects of concealment on nest survival may be difficult to detect if strong selection for concealed nest sites canalizes variation among nests such that most occur in “adaptive peaks” providing adequate concealment (Latif, Heath, & Rotenberry, 2012; Remeš, 2005). However, even studies employing experimental removal of vegetation have returned mixed support for the nest concealment hypothesis (e.g., Bengtson, 1972; Howlett & Stutchbury, 1996; Latif et al., 2012; Peak, 2003). Numerous intrinsic and extrinsic factors may influence the effect of concealment on nest success. For example, birds with more brightly colored plumage appear more dependent on vegetation to conceal the nest from predators (Borgmann & Conway, 2015), and the benefits of visual concealment may depend on the composition of the local predator community (Clark & Nudds, 1991; Colombelli‐Negrel & Kleindorfer, 2009; Dion, Hobson, & Lariviere, 2000). More problematic, however, are methodological aspects of studies that produce biased inference with regard to effects of concealment on nest survival (Borgmann & Conway, 2015; Burhans & Thompson, 1998; Gibson, Blomberg, & Sedinger, 2016; McConnell, Monroe, Burger, & Martin, 2017). Here, we focus on a recently highlighted methodological bias pervasive in research regarding habitat–fitness relationships in greater sage‐grouse (*Centrocercus urophasianus*).

The greater sage‐grouse (hereafter, sage‐grouse) is a precocial, ground‐nesting species of conservation concern inhabiting sagebrush ecosystems of western North America. Although sage‐grouse nest beneath shrubs—primarily sagebrush—perennial grasses and forbs in the interspaces between shrubs have long been thought to provide critical concealment of nests from potential predators (Connelly, Schroeder, Sands, & Braun, 2000). This hypothesis is supported by studies reporting positive associations between height and/or cover of herbaceous vegetation surrounding nest sites and nest survival (Coates & Delehanty, 2008; DeLong, Crawford, & DeLong, 1995; Doherty et al., 2014; Gregg, Crawford, Drut, & DeLong, 1994; Sveum, Edge, & Crawford, 1998). Consequently, sage‐grouse conservation efforts and land management policy have focused on increasing herbaceous hiding cover in suitable nesting habitat throughout the range of the species. Although direct links between livestock grazing and sage‐grouse demography are lacking, studies indicating positive effects of herbaceous vegetation height and/or cover on nest survival provide a plausible mechanism linking livestock grazing and nest success (Connelly & Braun, 1997; Connelly et al., 2000), a key demographic rate for sage‐grouse (Taylor, Walker, Naugle, & Mills, 2012). Thus, the validity of inference about the importance of herbaceous hiding cover for sage‐grouse nest success has major implications for the management of sagebrush ecosystems, where livestock grazing is a ubiquitous land use (Knick et al., 2003).

Recent evidence has demonstrated that the positive association between grass height, a commonly used metric of herbaceous concealing cover among sage‐grouse nesting studies, and nest survival may be indicative of biased methods rather than a causal relationship (Gibson, Blomberg, et al., 2016; McConnell et al., 2017). Using both empirical and simulation approaches, it has been shown that measuring grass height at nests following nest fate (i.e., hatch or failure) produces inflated or even spurious statistical relationships between grass height and nest survival. Because successful nests persist and are therefore measured later in the season than failed nests, measured concealment is greater at successful nests due to concurrent plant growth rather than a presumed reduction in predation. Despite knowledge of this sampling issue dating back decades (e.g., Burhans & Thompson, 1998), this sampling bias remains pervasive in sage‐grouse and other ground‐nesting bird literature, with a majority of sage‐grouse studies sampling vegetation following nest fate (Gibson, Blomberg, et al., 2016).

Given the far‐reaching implications derived from inference about grass height and sage‐grouse demography, we were interested in exploring the generality of recent findings reported by Gibson, Blomberg, et al. (2016), and McConnell et al. (2017). Using field data from four geographically distinct study sites representative of the diversity of vegetation communities, predator communities, precipitation regimes, and evolutionary history of grazing found across the range of sage‐grouse, we tested the hypothesis that studies using biased field methods that had previously supported a positive association between grass height measured around the nest and nest survival would fail to support such an association after accounting for phenology.

## 2 METHODS

We employed the model‐based methods presented in Gibson, Blomberg, et al. (2016) to correct for phenology in a reanalysis of three datasets from Montana, Utah, and Wyoming (Table 1). In a dataset from Eureka County, Nevada, analyzed by Gibson, Blomberg, et al. (2016), vegetation measurements were made at predicted hatch date and a linear regression relating vegetation height to the date of measurement was used to predict vegetation height at fate date, thereby demonstrating the potential bias arising from such a sampling scheme. We employed this concept in reverse fashion, that is, we regressed vegetation height on date of measurement to predict grass height at hatch date, as although it had been sampled using unbiased methods.

**Table 1:** We used predictions from five studies across the range of greater sage‐grouse, representing *n* = 1204 nests over a total of 24 study site‐years

Study area	*n*	Years	Transect length (m)	Samples per nest	Data source
Eureka County	396	2004‐2012	10	10	Gibson, Blomberg, et al. (2016);
PRB North	209	2003‐2006	30	20	Doherty et al. (2014)
PRB South	174	2004‐2006	30	20	Doherty et al. (2014)
Roundup	320	2012‐2015	12	8	J. Smith, Unpublished Data
NE Utah	105	2012‐2015	30	20	S. Dettenmaier, Unpublished Data
Total	1204				

Each study sampled grass height similarly, using measurements of the nearest grass height to various points along two intersecting transects centered at the nesting shrub. However, total transect length and the number of samples per nest varied by study.

### 2.1 Datasets

Reanalyzed datasets included a previously published study that found a significant positive influence of live grass height on sage‐grouse nest survival across two study areas in the Powder River Basin (PRB) in southeast Montana (hereafter PRB North, *n* = 209) and northeast Wyoming (hereafter PRB South, *n* = 164; Doherty et al., 2014); preliminary data from an ongoing evaluation of grazing treatments on sage‐grouse ecology in central Montana (Joseph Smith, University of Montana, Unpublished Data, *n* = 320); and the first 4 years of a study comparing sage‐grouse demography across two study areas in northern Utah (Seth Dettenmaier, Utah State University, Unpublished Data, *n* = 105). Including findings from Gibson, Blomberg, et al. (2016), these studies encompassed 1204 sage‐grouse nests over 24 study site‐years from across the range of sage‐grouse (Table 1). Each study used similar methodologies to sample herbaceous vegetation surrounding nest sites by taking multiple measurements of grass height along intersecting transects centered on the nesting shrub and using the mean of replicated measurements to represent grass height‐surrounding nests (Table 1).

### 2.2 Statistical analyses

We assumed hatch date was 27 days after the estimated nest initiation date and applied a correction to measured grass height covariates following Gibson, Blomberg, et al. (2016):

$$\begin{array}{cc} & {\text{GrassHeight}}_{\mathrm{Hatch}}=\\ & {\text{GrassHeight}}_{\mathrm{Fate}}-\left({\text{SurveyDate}}_{\mathrm{Fate}}-{\text{SurveyDate}}_{\mathrm{Hatch}}\right)\times \beta_{\mathrm{grass}} \end{array}$$

where, for each study area and year, we fit a linear regression of measured grass height (GrassHeight_Fate_) on day of nesting season (SurveyDate_Fate_) to estimate β_grass_. This simple correction provided a standardized measurement for grass height across nests regardless of fate. We estimated the effect of grass height on nest success using both corrected and uncorrected covariate measurements by fitting Bayesian daily nest survival models to each dataset (Schmidt, Walker, Lindberg, Johnson, & Stephens, 2010) with the exception of data from Gibson, Blomberg, et al. (2016), who provided estimates from their published analysis. In this approach, we estimated nest survival (*S*) for each nest (*i*) on each day of the nesting season (*t*) via a logit‐linear model, which at minimum included an intercept (β_0_) and coefficient for grass height, while also including coefficients that respective authors deemed supportive in top models. Nest encounter histories consisted of observed nest states (*y*) for each day of observation, where *y*_*i,t*_ = 1 if nest *i* was observed alive on day *t*,* y*_*i,t*_ = 0 if nest *i* was observed to have failed (female absent and some or all eggs destroyed), and *y*_*i,t*_ = NA on days when nest state was not observed. Beginning on the first day after the nest was detected,

$$y_{i,t}∼\text{Bern}\left(y_{i,t-1}S_{i,t}\right)$$

and

$$\text{logit}\;\left(S_{i,t}\right)=\beta_{0}+x_{i}{}^{\prime }\beta$$

Specifically, Doherty et al. (2014), following the original population analyses in Walker (2008), modeled nest survival using covariates including a main and quadratic effect for nest age, and categorical variables for a particularly harsh spring nesting season with major snow events that caused nest abandonment (2003) and the two study regions (PRB North and PRB South). Although the PRB datasets were collected independently, they were combined in the analysis presented in Doherty et al. (2014), and we combine them here for consistency. Although it appears this study was mistakenly recorded as having used a fate date protocol in Gibson, Blomberg, et al. (2016; Table 1), the investigators did attempt to control for phenology by sampling vegetation near the predicted hatch date regardless of nest fate. Nonetheless, close examination of the dataset revealed that a temporal bias in measurement date existed across all study site‐year combinations, such that successful nests were measured from 2 to 10 days later than failed nests, on average. To attempt to correct this persistent bias and maintain consistency among reanalyzed datasets, we corrected grass heights to predicted hatch date in the PRB North and PRB South datasets, but these corrections were generally smaller than corrections in the other reanalyzed datasets. Unpublished data from J. Smith included covariates for the log of distance to major roads and a measure of 4‐day cumulative rainfall, as well as a random effect for year. Data from Gibson, Blomberg, et al. (2016), and models fit to Utah data included only an intercept and coefficient for measurements of grass height. Our estimates of daily nest survival and nest success are only reflective of the incubation period, as sage‐grouse nests are typically found after the onset of incubation, and thus overestimate true nest success from initiation to hatch (Blomberg, Gibson, & Sedinger, 2015). Moreover, as monitoring intensity of prenesting females may have varied among datasets, incubation success may be more or less biased relative to true nest success and overall success rates are therefore not directly comparable among studies.

We fit daily nest survival models in JAGS 4.0 (Plummer, 2003) with the package rjags (Plummer 2016) in R 3.3.0 (R Core Team 2016), estimating posterior distributions with a total of 90,000 samples from 3 independent Markov chain Monte Carlo (MCMC) chains (30,000 per chain) after discarding the first 20,000 iterations from each chain for burn‐in. We placed vague normal prior distributions on all coefficients (μ=0; σ=1000). Using coefficient posterior distributions, we generated predictions for the mean influence of grass height on nest success, the product of daily nest survival over a 27‐day incubation period, and 95% credible intervals over the range of grass height values observed within each respective dataset. We held additional covariates at their mean value where applicable.

We performed an additional analysis to provide a comprehensive assessment of the influence of grass height on nest survival across datasets, excluding nests from Eureka County for which we only had data on the predicted response. Here, we pooled datasets and used generalized linear mixed models to test whether grass surrounding successful nests was taller than grass surrounding failed nests after accounting for phenology. Under the null hypothesis, grass heights (GH) measured at nests are a linear function of ordinal date of measurement (DAY; days since January 1), with normally distributed errors and no difference between successful and failed nests. Our alternative hypothesis was that grass is taller at successful nests than at failed nests after accounting for the linear function of ordinal date. We first used AIC_C_ model selection (Burnham & Anderson, 2002) to determine the best structure for a null (i.e., phenology) model. We considered a phenology model with a random intercept for each study area‐year (1|STUDY:YEAR) combination to allow for variation in grass height inherent among geographically distant study areas and in different years, and a random intercepts and slopes phenology model (DAY|STUDY:YEAR) to allow for different rates of grass growth among years and study areas. To aid in model convergence, we centered the independent variable DAY by subtracting the median day of measurement from all observations. After we determined the best structure for the phenology model using AIC_C_, we used a likelihood ratio test to assess support for our alternative hypothesis, which was represented with a model following the structure of the most supported phenology model and including a categorical fixed effect for nest fate (FATE; failed = 0, hatched = 1). Linear mixed models were fit using the lme4 package (Bates, Maechler, Bolker, & Walker, 2015) in R. Using these datasets, we also tabulated all corrected grass height measurements at successful and failed nests and performed a one‐sided Kolmogorov–Smirnov test to examine if distributions of measurements differed between pooled data sets. A one‐sided test was chosen to increase statistical power given our a priori expectation that grass would be taller surrounding successful nests than failed nests.

## 3 RESULTS

**Figure 1:**
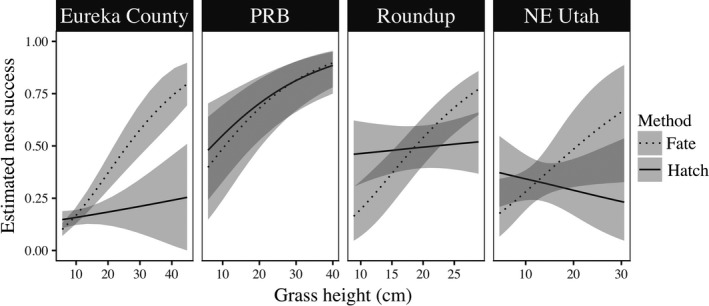
Predicted response of sage‐grouse nest success (and 95% CI [Eureka County] or CRI [other studies]) to live grass height using measurements collected with a biased method following determination of nest fate (dotted lines), and those measured or corrected to the predicted hatch date of nests (solid lines). Nest data includes studies from the powder river basin (PRB) in southeastern Montana (PRB North, Doherty et al., 2014, *n* = 209, 2003–2006) and northeast Wyoming (PRB South, Doherty et al., 2014, *n* = 174, 2004–2006); Eureka County, Nevada (Gibson, Blomberg, et al., 2016, *n* = 396, 2004–2012); central Montana near the town of Roundup (J. Smith, University of Montana, unpublished data, *n* = 320, 2012–2015), and northeast Utah (Dettenmaier, Utah State University, unpublished data; *n* = 105, 2012–2015). Note that limits of x‐axes change to reflect the range of grass heights observed within respective studies

Uncorrected, each of the three reanalyzed datasets revealed a strong, positive association between grass height and daily nest survival (Figure 1; dotted lines). Estimated coefficients for grass height using uncorrected grass heights were 0.063 (95% CI from 0.037 to 0.092) for PRB North and PRB South, 0.099 (95% CI from 0.063 to 0.137) for Roundup, and 0.058 (95% CI from 0.002 to 0.118) for NE Utah. Corrections to measured grass heights averaged—1.32 cm and mean absolute correction (|corrected–uncorrected|) was 2.08 cm, with a standard deviation of 2.31 cm. Following adjustment of measured grass heights to remove temporal bias, we found no association between grass height and nest survival in two of the three datasets (Roundup and NE Utah), and a weakened but persistent association in the PRB dataset (Figure 1; solid lines). Estimated coefficients for grass height using corrected grass heights were 0.053 (95% CI from 0.025 to 0.081) for PRB North and PRB South, 0.008 (95% CI from ‐0.027 to 0.042) for Roundup, and −0.015 (95% CI from −0.060 to 0.032) for NE Utah.

**Figure 2:**
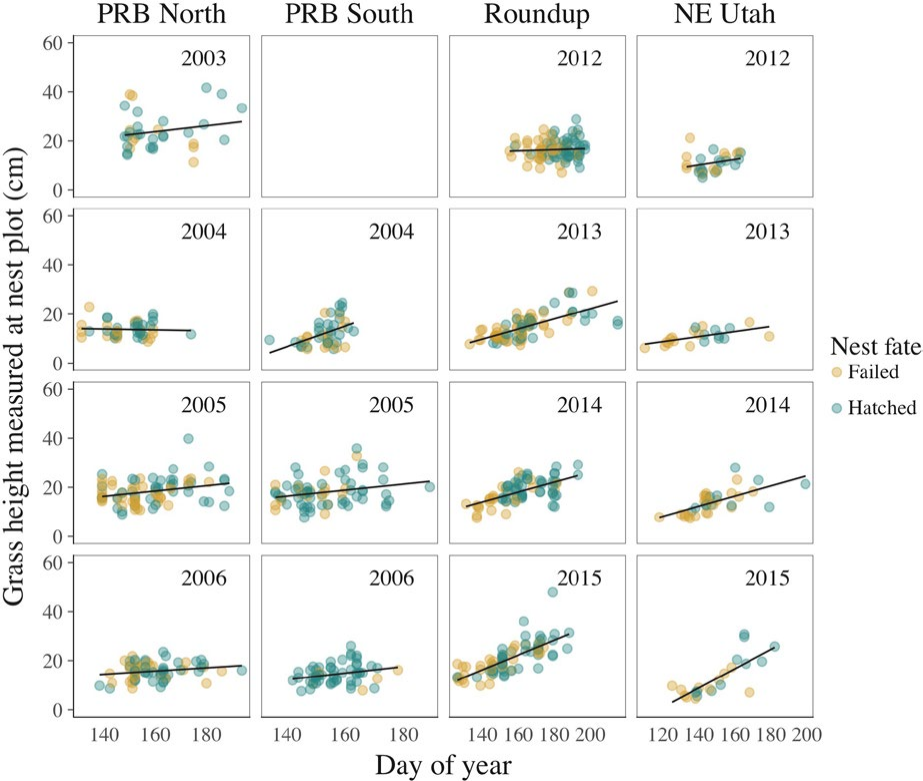
Average grass height surrounding successful and failed sage‐grouse nests (*n* = 808) at the ordinal date of measurement by year (rows) and study area (columns). After accounting for phenology, a difference in grass height between successful and failed nests was not supported

**Figure 3:**
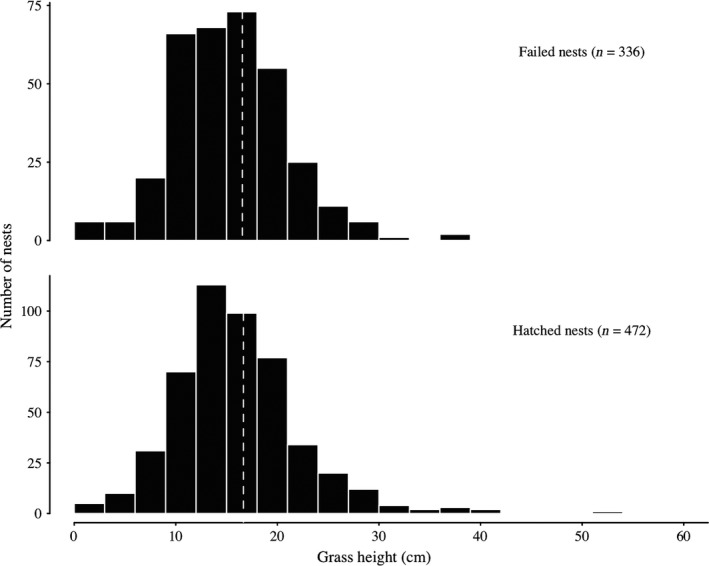
Grass heights surrounding greater sage‐grouse nests (*n* = 808) corrected to hatch date. Median height of grass‐surrounding nests (dashed vertical lines) was 15.26 cm at successful nests and 15.14 cm at failed nests. A one‐sided Kolmogorov–Smirnov test provided no evidence that the distributions of grass heights differed between successful and failed nests (ground‐nesting *p* = .307)

The random intercept and slope phenology model (conditional *R* ^2^ = 0.51 [Nakagawa & Schielzeth, 2013]) received the most support with an AIC_C_ score 9.64 units lower than the constant slope model (conditional *R* ^2^  = .46) and was used as the null model (Figure 2). The alternative hypothesis, that grass height surrounding successful nests was greater than that surrounding failed nests after accounting for phenology, was not supported (χ^2^ = 2.74, *df* = 1, *p* = .098). Overall, median height of live grasses, corrected to hatch date, was 15.3 cm at successful nests (*n* = 336) and 15.1 cm at failed nests (*n* = 472; Figure 3). A one‐sided Kolmogorov–Smirnov test provided no evidence that the distributions of phenology‐corrected grass heights differed between successful and failed nests when pooling across sites and years (*p* = .307).

## 4 DISCUSSION

While our analyses revealed mixed support for relationships between grass height and nest survival in sage‐grouse, they confirmed recent findings that associations between herbaceous vegetation structure and nest success are frequently byproducts of temporally biased sampling rather than indicative of effect of concealing cover on detectability by predators (Gibson, Blomberg, et al., 2016; McConnell et al., 2017). Sampling vegetation following nest fate, a pervasive practice in studies of sage‐grouse and other ground‐nesting birds, consistently produces spurious relationships between grass height and nest survival and should, therefore, be avoided. As field crews are rarely able to strictly adhere to a schedule due to weather or other logistic constraints, even studies using field protocols intended to control for phenology may be affected by some degree of temporal bias between failed and successful nests, producing inflated effect sizes (e.g., the PRB dataset reanalyzed here; Doherty et al., 2014).

Taller grass may be associated with reduced nest predation under some conditions, such as in the context of particular predator communities or in years with particularly tall grass. However, grass height does not appear to be a universal indicator of nesting habitat quality for sage‐grouse. Including the PRB dataset, we are aware of only three published studies using unbiased methods that support a positive association between grass height and nest survival (Doherty et al., 2014; Gregg et al., 1994; Sveum et al., 1998) among the 11 published studies testing for such an effect (Table 1 in Gibson, Blomberg, et al., 2016). Although the results have generally been interpreted to support the hypothesis that taller grass promotes greater nest survival (Connelly et al., 2000; Crawford et al., 2004), data presented by Sveum et al. (1998; Table 2) merely indicated that cover of short grasses (<18 cm) was lower at successful nests than failed nests in 1 out of 2 years (*n* = 32 nests), while cover of tall grasses (≥18 cm) did not differ between successful and failed nests in any year, even using a liberal α level of 0.1. Positive relationships between grass height and nest survival may, in fact, be uncommon. It is telling that, when analyzed together, data from the four study areas examined here provided no evidence for a difference in herbaceous vegetation height between successful and failed nests after accounting for plant phenology and timing of sampling (Figures 2 and 3).

The research and management communities must guard against uncritical acceptance of intuitive but untested mechanistic explanations for correlative patterns emerging from observational studies of habitat–fitness relationships. Within the sagebrush ecosystem, the broad acceptance that taller grass causes greater nest success by concealing nests from predators is an example of this type of untested logical connection, as equally plausible alternative hypotheses exist. For example, in multiyear studies, annual variation in precipitation and temperature in the prenesting and nesting periods may simultaneously affect female body condition, incubation behavior, and plant phenology. If conditions favorable to increased body condition or nest attentiveness have coincident positive effects on grass growth, nest success may be positively correlated with grass height absent any causal relationship between the two variables.

An experimental approach involving manipulation of vegetation height‐surrounding nests could circumvent these issues, but would be fraught with its own set of difficulties. Sage‐grouse females display a propensity toward abandoning reproductive efforts following disturbance by investigators (e.g., Gibson, Blomberg, Atamian, & Sedinger, 2015; Moynahan, Lindberg, Rotella, & Thomas, 2007). Disturbance from experimental manipulation at treatment nests would, therefore, need to be simulated at control nests such that observer‐induced abandonment rates would be equal among nests in both groups. This may present an ethical dilemma for a species of conservation concern, or may simply yield sample sizes with inappropriately low statistical power. Furthermore, results of such an experiment would be of questionable relevance to management if manipulations bore little resemblance to defoliation patterns arising via herbivory (France, Ganskopp, & Boyd, 2008). Thus, experimental research is unlikely to provide an easy resolution to the problem. A critical examination of past evidence and careful consideration of alternative mechanistic hypotheses are warranted when considering the observational evidence at hand.

Habitat–fitness relationships are often context‐dependent, and therefore variable across a species’ range. Effects of concealment on nest survival, for example, may be more likely where cover is sparse. If that were the case, we might expect effects of grass height on nest survival to be more common in study sites characterized by low‐shrub cover‐surrounding nests. Indeed, the positive association between grass height and nest survival in the PRB study site reanalyzed here occurred in the eastern portion of the range, characterized by high spring precipitation and herbaceous vegetation cover compared to the rest of the sage‐grouse range (Doherty, Evans, Coates, Juliusson, & Fedy, 2016). However, there was no relationship between grass height and nest survival in the Roundup study area, which had the lowest average shrub cover (18%) among datasets we considered. Selection of nest sites surrounded by tall grasses (Hagen, Connelly, & Schroeder, 2007) may result in a truncated covariate space such that nests surrounded by very short vegetation are rarely observed, thereby precluding the ability to detect an effect on survival (Chalfoun & Schmidt, 2012; Latif et al., 2012). However, with data from 15 study site‐year combinations, we are confident we have surveyed a representative range of conditions chosen by nesting females. The lack of difference in grass height between successful and failed nests across these datasets strongly suggests that height of grasses was not a limiting resource (Figure 3).

The absence of support for an effect of grass height does not imply concealment is wholly unrelated to nest survival in sage‐grouse. Selection for larger, taller sagebrush for nest substrates and preference for nesting in areas with greater areal cover of shrubs are well documented (reviewed in Hagen et al., 2007). In preferred sites, grasses and forbs may simply provide little additional visual or olfactory obstruction between a nest and a potential predator beyond that already provided by shrubs (see France, Ganskopp, & Boyd, 2008). Furthermore, while grasses and forbs afford mostly lateral cover, shrubs may provide more effective cover from aerial visual predators such as common ravens (*Corvus corax*), a primary nest predator for sage‐grouse (Coates, Connelly, & Delehanty, 2008; Coates & Delehanty, 2008). Previous research indicates nest site selection in sage‐grouse is driven by avian predators at broad scales (Dinkins, Conover, Kirol, & Beck, 2012) and characteristics of nest sites at small scales are more consistent with avoidance of visual (i.e., avian) predators than olfactory (i.e., mammalian) predators (Conover, Borgo, Dritz, Dinkins, & Dahlgren, 2010; Fogarty, Elmore, Fuhlendorf, & Loss, 2017). The lack of association between height of grasses and survival may also indicate a trade‐off between nest concealment and the ability of incubating females to detect predators from a distance and alter their behavior in such a way as to reduce detection (Götmark, Blomqvist, Johansson, & Bergkvist, 1995).

Nest success is only one among several influential vital rates affecting sage‐grouse population growth, and further research is needed to address how structure of grasses and forbs affects other life stages in sage‐grouse. Studies of other grouse suggest vegetation height may be an important driver of brood survival. For example, increased vegetation height and/or greater insect abundance resulting from reduced grazing intensity positively affected production in black grouse (*Tetrao tetrix*) in Britain (Baines, 1996; Calladine, Baines, & Warren, 2002). The positive effect on production was, however, diminished or even reversed when grazing reduction treatments covered larger areas (Calladine et al., 2002), suggesting mosaics of vegetation height may confer greater benefits than uniformly tall vegetation (also see Baines, Richardson, & Warren, 2017; Jahren, Storaas, Willebrand, Moa, & Hagen, 2016). Taller vegetation may also moderate thermal extremes experienced by grouse, a function which may take on increased importance under climate change (Hovick, Elmore, Allred, Fuhlendorf, & Dahlgren, 2014). Although selection of sites with greater visual concealment by brood‐rearing sage‐grouse has been documented (Kaczor, Herman‐Brunson, & Jensen, 2011; Schreiber et al., 2015), studies testing effects of herbaceous vegetation structure on sage‐grouse chick survival are few and have produced mixed results (Aldridge, 2005; Gregg & Crawford, 2009). Recently, Gibson, Blomberg, et al. (2016) found survival of sage‐grouse chicks to 2 weeks of age was positively associated with height of grasses surrounding the nest, presumably because structure of vegetation at the nest site is assumed to be correlated with structure of vegetation encountered by the precocial chicks during the first weeks of life. Again, however, a causal relationship between grass height and chick survival cannot be inferred. Positive relationships between herbaceous plant height and chick survival could implicate concealment from predators, but it is also plausible that taller grass at the nest is associated with some unmeasured factor—for example, site productivity, precipitation, or soil moisture—which in turn influences factors causally related to chick survival.

While the herbaceous understory is a key component of sagebrush ecosystems and sage‐grouse habitat (e.g., Chambers et al., 2014), its role in concealing nests from predators has been overstated in management guidelines and land management documents. For example, the habitat assessment framework (HAF; Stiver et al., 2015), a tool used by the US Bureau of Land Management and US Forest Service to evaluate whether public lands are meeting habitat requirements of sage‐grouse, included guidelines for maintaining a minimum height of perennial grasses and forbs in upland nesting habitat (18 cm) based largely on studies suggesting positive effects of vegetation height on nest success. There is, however, little evidence for the existence of the causal relationship between grass height and nest survival on which these guidelines were predicated. While it appears these “fourth order” guidelines may place unwarranted emphasis on the importance of maintaining herbaceous hiding cover for nesting, it should be noted that the HAF appropriately lays out a hierarchical management approach which suggests policies be set at the rangewide and regional scales to limit habitat loss and fragmentation—known causes of population declines among prairie grouse—but emphasizes that significant flexibility should be granted to local managers applying finer scale guidelines (see Chapter 1, Stiver et al., 2015). Persistent, broad‐scale threats to sagebrush ecosystems including oil and gas development (Naugle, Doherty, Walker, Holloran, & Copeland, 2011), wildfire and invasive annual grasses (Coates et al., 2016), cropland conversion (Smith et al., 2016), and conifer encroachment (Miller, Naugle, Maestas, Hagen, & Hall, 2017) are well‐documented drivers of sage‐grouse population declines and should therefore be the highest priority for managers. Maintenance of tall grasses and forbs for nesting cover should not distract managers from addressing these larger threats or preclude the use of management tools that could otherwise improve sage‐grouse habitat.

## AUTHOR CONTRIBUTIONS

JTS conceptualized the study, collected field data in central Montana, compiled and quality checked data from all study sites, analyzed data, produced figures, and wrote the manuscript. JDT analyzed data, produced figures, and assisted in writing the manuscript. KED collected field data in PRB and assisted in writing the manuscript. BWA, JDM, and DEN assisted with study conceptualization, interpretation of results, and manuscript writing, and revised several early versions of the manuscript. LIB and TAM contributed field data in central Montana and Northern Utah, respectively, and critically revised the final manuscript. SJD collected field data in Northern Utah. All authors critically revised and approved the final version of the manuscript.
